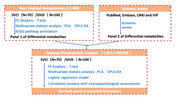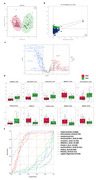# Serum non‐targeted metabolomic and lipidomic analyses reveal potential biomarkers to predict cognitive impairment for subcortical ischemic vascular disease

**DOI:** 10.1002/alz.087895

**Published:** 2025-01-09

**Authors:** Yunsi Yin, Yifei Zhang, Fan Wang, Yi Tang, Qi Qin

**Affiliations:** ^1^ Department of Neurology & Innovation Center for Neurological Disorders, Xuanwu Hospital, Capital Medical University, National Center for Neurological Disorders, Beijing, China, Beijing, Beijing China; ^2^ New York University, New York City, NY USA; ^3^ Harbin Medical University, Harbin China; ^4^ Department of Neurology & Innovation Center for Neurological Disorders, Xuanwu Hospital, Capital Medical University, National Center for Neurological Disorders, Beijing, Beijing China; ^5^ Neurodegenerative Laboratory of Ministry of Education of the People’s Republic of China, Beijing, Beijing China

## Abstract

**Background:**

It is challenging to distinguish which subcortical ischemic vascular disease (SIVD) patients will present with cognitive impairment. A blood‐based biomarker to distinguish SIVD patients with cognitive impairment would be superior to neuropsychological measures and neuroimaging measures in terms of cost, time, and feasibility for repeated measures. Metabolomics profiling studies could help identify blood‐based biomarkers for SIVD patients with cognitive impairment.

**Method:**

100 SIVD patients without cognitive impairment,70 SIVD patients with cognitive impairment, 30 DLB patients and 30 AD patients were recruited from three Chinese research hospitals. 34 SIVD patients without cognitive impairment and 34 SIVD patients were follow‐up for 2 years. Patients’ serum samples were assessed using non‐targeted metabolomics and lipidomics analyses.

**Result:**

Six metabolites including TG(52:2)‐FA18:1, DHCer(d18:0_24:0), ChE(22:4), Cer(d18:1_18:0), SM(d18:1_26:0) and Hex2Cer(d18:1_24:0) were identified as informative biomarkers for cognitive impairment in SIVD patients. The performance was stable after 2 years follow up and in differential diagnosis among AD and DLB patients.

**Conclusion:**

Our study has developed an accurate and accessible means based on blood samples to identify SIVD patients at high risk of cognitive impairment. Dysregulation of sphingolipid metabolism was involved in the neurobiological pathways that contributed to cognitive impairment in SIVD patients.